# Capturing the Interplay between Risk Perception and Social Media Posting to Support Risk Response and Decision Making

**DOI:** 10.3390/ijerph18105220

**Published:** 2021-05-14

**Authors:** Huiyun Zhu, Kecheng Liu

**Affiliations:** 1 School of Management Science and Engineering, Nanjing University of Information Science & Technology, Nanjing 210044, China; 2 Shanghai Engineering Research Center of Finance Intelligence and Institute of Fintech, Shanghai University of Finance and Economics, Shanghai 200434, China; k.liu@henley.ac.uk; 3 Informatics Research Centre, University of Reading, Whiteknights, Reading RG6 6UD, UK

**Keywords:** risk perception, social media posting, Granger causality analysis, impulse response functions, social media

## Abstract

This research aims to capture the interplay between risk perception and social media posting through a case study of COVID-19 in Wuhan to support risk response and decision making. Dividing users on Sina Weibo into the government, the media, the public, and other users, we address two main research questions: Whose posting affects risk perception and vice versa? How do different categories of social media users’ posts affect risk perception and vice versa? We use Granger causality analysis and impulse response functions to answer the research questions. The results show that from one perspective, the government and the media on Sina Weibo play critical roles in forming and affecting risk perceptions. From another perspective, risk perception promotes the posting of the media and the public on Sina Weibo. Since government’s posting and media’s posting can significantly enhance the public’s perceptions of risk issues, the government and the media must remain vigilant to provide credible risk-related information.

## 1. Introduction

Although experts conduct risk assessments for hazards, most people rely on intuition to make risk judgments, which is named “risk perception” [1]. Risk perception is commonly defined as “the subjective judgment that people make about the characteristics and severity of risk”, often associated with disasters [2]. Understanding risk perceptions of a public health crisis is critical for disease prevention and control [3] because risk perception determines which hazards people care about and how they deal with them [4].

Mass media have long been considered to be vital shapers of the public’s risk perceptions. The media can function as a “social amplification station” to form the social experience of risk, by either amplifying or attenuating public risk perception [5]. However, the emergence of Web 2.0 poses a challenge to the traditional communication model. With the capability of reaching out to a broad audience and interacting instantly, the use of social media facilitates the dissemination of health-related content and information [6]. Especially when it comes to risk issues, social media can profoundly affect people’s risk perception, more than legacy media [3]. Social media is an important bridge and channel for risk perception transmission, and it is also an important amplifying station for social risks.

Public perceptions of risk can be influenced by, e.g., the amount, valence, and tone of media coverage [4,7]. The exposure and consumption of information during pandemic outbreaks may alter people’s risk perception and trigger behavioral changes, which can ultimately affect the evolution of the pandemic, especially in the early stages [7].

Social media coverage is correlated with the level of risk perception [3]. However, there are many different types of users, such as the government, the media, and the public, and no studies to date have directly examined the relationship between different users’ posting and risk perception. Are there any differences in the relationship between different users’ posting and risk perception when a health risk emerges?

Using the case of Sina Weibo activity about COVID-19, we explore the interplay between risk perception and social media posting. To achieve this, we address two main questions:

RQ1: Whose posting can affect risk perception and vice versa?

RQ2: How does social media users’ posting affect risk perception and vice versa?

To answer the research questions above, in this paper, we subdivide microblogs on Sina Weibo into categories based on the different sources, e.g., the government, the media, the public, and other users. Then, we use Granger causality analysis and impulse response functions to find the interplay between different users’ posting and risk perception.

## 2. Literature Review

### 2.1. Risk Perception

Laypeople evaluate risks mostly according to subjective perceptions, intuitive judgments, and inferences from media coverage, often with limited information [4]. This is named “risk perception”. Slovic divides risk into dread risk and unknown risk based on people’s intuitive feelings; dread risk represents the uncontrollability and severity of the risk, while unknown risk represents the uncertainty of the risk [1].

Risk perception is important in health and risk communication because it determines which hazards people care about and how they respond to them [4]. Improving our understanding of risk perception is important so that we may improve our capabilities when it comes to communication, decision support, and management [8].

The major approach of measuring risk perception is the psychometric paradigm, using questionnaire survey methods to measure the perception of different risk events [7,9,10,11,12,13]. However, risk perception obtained by this method is static, and the values may be inaccurate due to the influence of people’s memory. Some researchers evaluate the public’s risk perception using online information search volume, such as with Google Trends or Baidu Search Index [14,15,16], which can scientifically and dynamically measure and evaluate risk perception.

Understanding the determinants of risk perception is essential for disseminating appropriate public health policy information. The main influencing factors of public risk perception are media [3,17,18], emotions (such as negative emotion, fear and anger) [13,19,20], socio-cultural aspects [21], etc. The review reveals that personal experience of a natural hazard and trust—or lack of trust—in authorities and experts have the most substantial impact on risk perception [22]. Socio-demographic variables such as gender, age, and income are rarely the main focus of contemporary risk perception research; instead, they are used mainly as control variables [8].

The public’s risk perception may affect their information search and sharing behavior. Analysis reveals a statistically strong relationship between risk perception and information-seeking efficacy on floods [2]. Xu et al. [23] find that perceptions of dread risk have a dominant and immediate impact on social networking site (SNS) sharing behavior in the buildup, breakout, and termination stages of emerging infectious disease (EID) events; perceptions of unknown risk have a dominant and persistent impact on sharing behavior in the abatement stage.

### 2.2. The Role of Media

The social amplification of risk framework (SARF) believes that the media plays a key role in the process of the public’s response to risk [24]. Traditional media is an important source of information that can influence how people perceive and respond to disaster events [25]. In recent years, scholars in related fields have paid attention to the role of social media platforms in the amplification or attenuation of risk perceptions. With the capability to reach out to a broad audience, the use of social media facilitates the dissemination of information [5]. Social media also allows individuals to participate in shaping risk perception through more immediate and accessible discussion of risk topics [26]. Therefore, social media becomes a major amplifying station for disasters and health-related hazards, and it is also an important bridge and channel for health-related risk communication. Social media channels complicate the way that risk is negotiated and communicated and have transformed the media landscape within which the original SARF was conceived, intensifying the challenges for risk communication as well as creating new opportunities [25].

A variety of media factors have been found to affect the public’s risk perceptions, such as amount, valence, and tone of media coverage and media channels and types [4]. Public attention is mainly driven by media coverage, and the exposure and consumption of information may alter people’s risk perception and trigger behavioral changes [6].

Different media sources have different impacts on risk perception. Television, interpersonal communication, and the category of miscellaneous online sources are significant regressors of the perceived health-related risk of a nuclear accident [12]. Personal risk perception was enhanced more by reported attention to international coverage, reduced by certain reported website attention, but enhanced by reported attention to public health agency websites during the Zika outbreak [11]. The research results of Yoo et al. [27] indicate that content-oriented social media may be more effective in influencing risk perception. They find that content-oriented social media exposure is significantly related to personal and social risk perception; however, user-oriented social media exposure does not affect risk perception [27].

Media factors may trigger behavioral changes, which can ultimately affect the evolution of the hazard. The legacy media coverage is correlated with the level of protective behaviors during emerging health threats [3]. Media exposure increases healthier behaviors in the use of public spaces in the case of the COVID-19 outbreak [28]. Social media use can significantly increase preventive behaviors via the public’s risk perception during infectious disease outbreaks [13].

### 2.3. Social Media Data

Social media has played a significant role in disaster management. It enables the general public to report incidents related to disaster events [29]. When a disaster occurs, social media users generate massive amounts of data on social media platforms such as Facebook and Twitter. These social media data with temporal and spatial attributes have become an important means of understanding public behavior [30]. Managers and researchers can analyze social media data for disaster detection [31,32], risk communication [12,33,34,35,36], intelligent decision-making [37], and emergency response [38,39]. For example, government social media can be used for increasing vigilance and awareness in the prodromal stage, disseminating information and increasing transparency in the acute stage, and focusing on mental health support and recovery policies in the chronic stage [40].

However, there are many challenges in acquiring and extracting hazard-related information from social media, including massive volume, unstructured data sources, signal-to-noise ratio, ungrammatical and multilingual data, and fraudulent message identification and removal [29]. In particular, the “filter bubbles” phenomenon brought about by the algorithm mechanism cannot be ignored. Web sites are now increasingly personalized—based on users’ web history, they filter information to show the content they think users want to see [41]. Subsequently, the subjective information push often keeps many valuable and diverse pieces of information out of the bubbles. In response to this threat, researchers and practitioners have developed algorithms and digital tools to combat such biased filter bubbles. For example, Bozdag and Van Den Hoven [42] show how norms are applied in two democracy models to fight against the filter bubbles.

### 2.4. Social Media Posting and Risk Perception

Previous studies have investigated the relationship between social media posting and risk perception in diverse ways (Table 1). The terms used in these papers are not consistent (for example, social media coverage [3] and social media sharing behavior [23]).

There is controversy about the causal relationship between risk perception and social media posting. Chan et al. [3] suggest that changes in the volume of information in social media (i.e., Twitter) are followed by different changes in risk perception. However, Xu et al. [23] find that changes in risk perceptions are followed by changes in social media sharing behavior. We further analyze the interplay between risk perception and social media posting in this paper to identify the Granger causality between them.

The measurements of risk perception used in Table 1 are obtained through questionnaires [3,35,43] and social network big data [23], while the data of social media posting are obtained through another set of questionnaires [35,43] and social media data [3,23].

A closely related term to social media posting is social media exposure [17,26,27]. Social media exposure is exposure to news and information about risk on social media, usually measured by asking how often respondents were exposed to news and information about risk on social media [44]. Social media exposure was positively related to forming risk perceptions [17].

## 3. Materials and Methods

### 3.1. Social Media Data

In the case study, we chose Sina Weibo as the data source. Sina Weibo is a popular social media site in China and is a Chinese equivalent to Twitter. Among all types of Weibo services in China, Sina Weibo is the most visited social media website.

We chose the social media data about COVID-19 in Wuhan from 25 January 2020 to 26 April 2020 as the research object. In December 2019, the Wuhan Center for Disease Control and Prevention detected cases of pneumonia of unknown cause. On 20 January 2020, academician Nanshan Zhong appeared live on CCTV’s prime time news program, emphasizing that the new crown virus can spread from person to person, requiring the public to be highly vigilant. The Chinese government upgraded the level of prevention and control. On 23 January 2020, Wuhan was completely locked down, and the airport and railway stations were closed. On 26 April 2020, Wuhan COVID-19 cases were cleared as declared by the authority. The period we chose covered the entire life cycle of the COVID-19 epidemic in Wuhan.

We executed data collection in two steps to crawl relevant data. The first step was to crawl the microblogs on Sina Weibo of COVID-19 in Wuhan from 25 January 2020 to 26 April 2020. The data were collected under the compound strategy of keywords, locations, and time, using the advanced search function of Sina Weibo. The search keyword we set was “Xin Guan Fei Yan” in Chinese characters, which means COVID-19, and the geographical location was limited to Wuhan. We set the type of post to the original microblogs. The crawled content included user name, publishing time, content, post hypertext reference, post identification (ID), and user hypertext reference.

In order to obtain as much data as possible and avoid the risk of search results being distorted by search engine algorithms, we applied two strategies. One was to use multiple Sina Weibo accounts and randomly select one account use each time for crawling. The second was to use the advanced search function of Sina Weibo and set the search conditions to be as detailed as possible. The crawling interval was set to every hour, and the search location was limited to Wuhan. In this way, we obtained as much data as possible to avoid the filter bubble.

The second step was to crawl user profiles according to user hypertext reference, so as to identify government, media, public, and other users. The users of Sina Weibo include the public and governments, companies, media, and other organizations. Government and media users are identified by industry categories in user profiles. We have observed that ordinary individuals are usually not authenticated. In this way, we regarded users with personal authentication and no authentication as the public. After information cleansing on Sina Weibo data, a total of 73,454 microblogs were obtained for our analysis in this article, involving 292 government users, 237 media users, 14,017 public users, and 661 other users. The descriptive statistics of social media data are shown in Table 2.

It can be seen from Table 2 that the public contributed the most microblogs, accounting for half of the total number of microblogs, but their average number of microblogs is the least at 2.25 per user. Although the number of media users is the smallest, their average number of microblogs is the largest at 122.30 per user. The government users have the lowest total number of microblogs.

### 3.2. Risk Perception Data

As described in Section 2, risk perception obtained by questionnaire survey method is static. This study aimed to obtain dynamic data on risk perception over time. Thus. we chose online information search volume as a dynamic indicator of risk perception.

**Figure 1:**
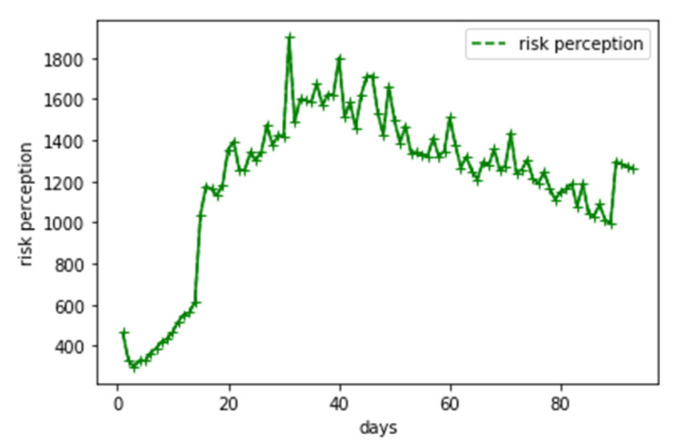
Time series of risk perception index.

Considering that our case was based on China’s national conditions, we used Baidu Search Index as the measure of risk perception (Figure 1). Similar to Google Trends, Baidu Search Index is based on the search volume of Internet users on Baidu, which is the most popular online search engine in China.

### 3.3. Granger Causality Analysis and Impulse Response Functions

We used Granger causality analysis and impulse response functions to answer the two research questions proposed in this paper. Granger causality analysis was conducted to determine whether the volume of different users’ posting in a previous day (i.e., with the time lag of one, two, or three days) was correlated with levels of risk perceptions in the current week, or vice versa. Impulse response functions were applied to find the impulse response between variables and answer the second research question. The variables involved are shown in Table 3.

For the purposes of our study, risk perception is represented by online information search volume, i.e., Baidu Search Index.

**Figure 2:**
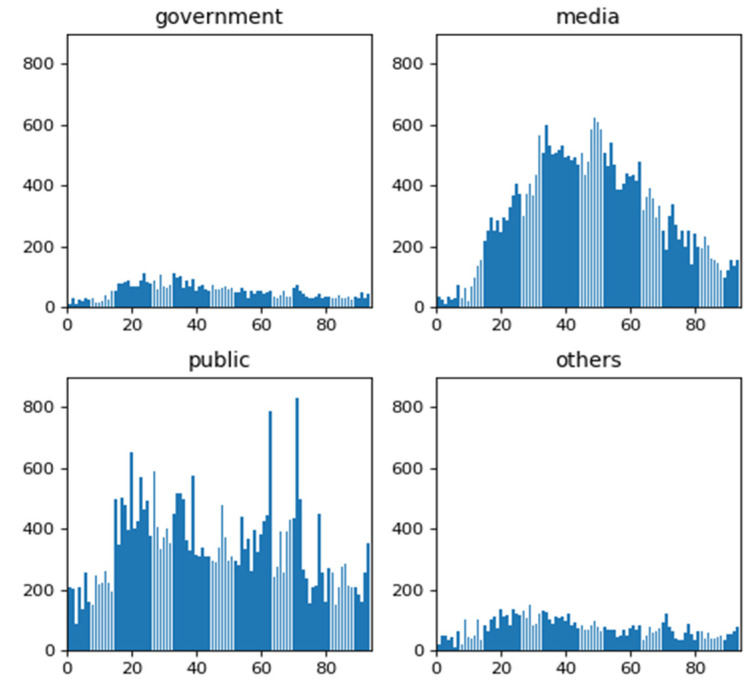
Time series of the volume of microblogs.

The postings by the government, the media, the public, and other users were measured by the volume of posting of different users. As mentioned in Table 2, the public and the media contribute the most microblogs, and the media has the highest average number of microblogs. The time series of the postings from the government, the media, the public, and other users is shown in Figure 2. It can be seen that the volume of microblogs shows roughly the same pattern as the risk perception index (i.e., Baidu Search Index); that is, it increases first and then decreases as the pandemic develops.

**Figure 3:**
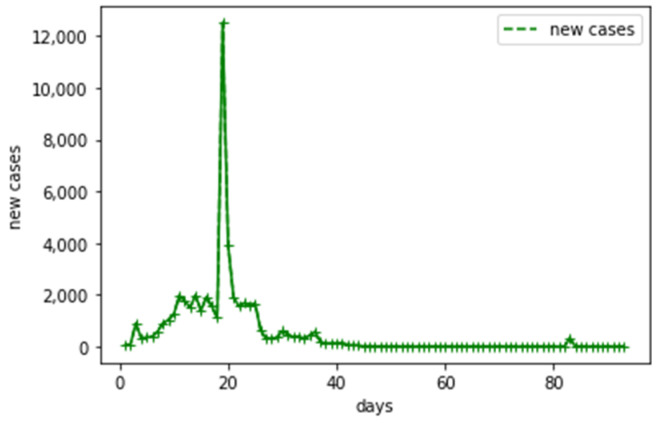
Time series of the number of new cases.

The number of cases, especially the number of new cases, is an important factor affecting risk perception during COVID-19 in Wuhan. We chose the number of new cases as an exogenous variable to include the impact of the number of new cases on risk perception in the model. The time series of new cases is shown in Figure 3. There is a huge outlier in the time series, which corresponds to 12 February 2020. On this day, the Chinese government changed its statistical standards to treat clinically diagnosed cases as confirmed cases, which resulted in a maximum in the number of new cases at this time.

We tested for the absence of Granger causality and found the impulse response between variables by the following VAR model:

(1) $$Perception_{t}=g_{0}+a_{1}Perception_{t-1}+\cdots +a_{p}Perception_{t-p}+b_{1}Posting_{t-1}^{k}+\cdots +b_{p}Posting_{t-p}^{k}+e_{1}NewCases_{t}+u_{t}$$

(2) $$Posting_{t}^{k}=h_{0}+c_{1}Posting_{t-1}^{k}+\cdots +c_{p}Posting_{t-p}^{k}+d_{1}Perception_{t-1}+\cdots +d_{p}Perception_{t-p}+e_{2}NewCases_{t}+v_{t}$$

Among them, k=0, 1, 2, 3, correspond to the government, the media, the public, and other users, respectively. Formula (1) represents Perception as a function of its own past value, the past value of $Posting^{k}$, an exogenous variable NewCases, and an error term. Formula (2) represents $Posting^{k}$ as a function of its own past value, the past value of Perception, an exogenous variable NewCases, and an error term. u, v are white noise. t is the index of a day.

Then, we tested H0 ($b_{1}=b_{2}=\cdots =b_{p}=0$) against HA ($Posting^{k}$ Granger causes Perception). Similarly, we tested H0 ($d_{1}=d_{2}=\cdots =d_{p}=0$) against HA (Perception Granger causes $Posting^{k}$). The lag length was determined according to the minimum values of AIC and BIC, and the VAR model was established on this basis.

## 4. Results

### 4.1. The Results of Granger Causality Tests

Whose posting can affect risk perception and vice versa? In addressing this research question, we use Granger causality analyses on time series data of the number of microblogs and Baidu Search Index.

Before Granger causality analysis and impulse response analysis, the stationary of the VAR model should be tested. If the VAR model is stable, Granger causality test and impulse response analysis make sense. After testing, the reciprocal values of all roots of the VAR model are less than 1, indicating that the structure of the VAR model is stable. Therefore, the conditions of Granger causality analysis and impulse response analysis are satisfied.

**Figure 4:**
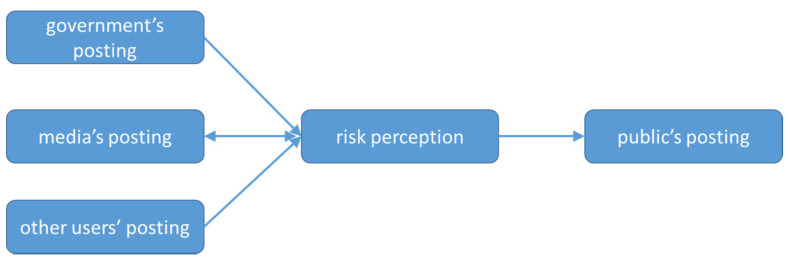
Causal channels of posting and risk perception.

The results of Granger causality tests between the government, the media, the public, and other users’ posting and risk perceptions are shown in Table 4 and Figure 4.

We found the existence of unidirectional causality from government’s posting to risk perception and from other users’ posting to risk perception, which means that changes in government’s posting and other users’ posting impact risk perception. The result of the unidirectional causality running from government’s posting to risk perception is consistent with the conclusion of Bec and Becken’s [45] proposal that the Twitter statements delivered by government agencies are found to influence risk perceptions.

Bidirectional causality was found between the media’s posting and risk perception in the case of Wuhan. The media users in this article include users who have registered as television (TV), radio, newspaper, magazine, media website, and new media on Sina Weibo. Bidirectional causality between media and risk perception shows not only that media coverage amplifies risk perception but also that the increase in risk perception promotes the increase in media coverage.

A unidirectional causality was found from risk perception to the public’s posting. This means that changes in risk perception affect the public’s behavior on social media.

Therefore, we may conclude that the government’s posting, the media’s posting, and other users’ posting are the Granger reason for the risk perception of COVID-19 in Wuhan. Risk perception is the Granger reason for the media and the public’s posting.

### 4.2. The Results of Impulse Response Functions

The relationship between social media users’ posting and risk perception is bidirectional, as discussed in the next subsections.

#### 4.2.1. Social Media Users’ Posting Affecting Risk Perception

**Figure 5:**
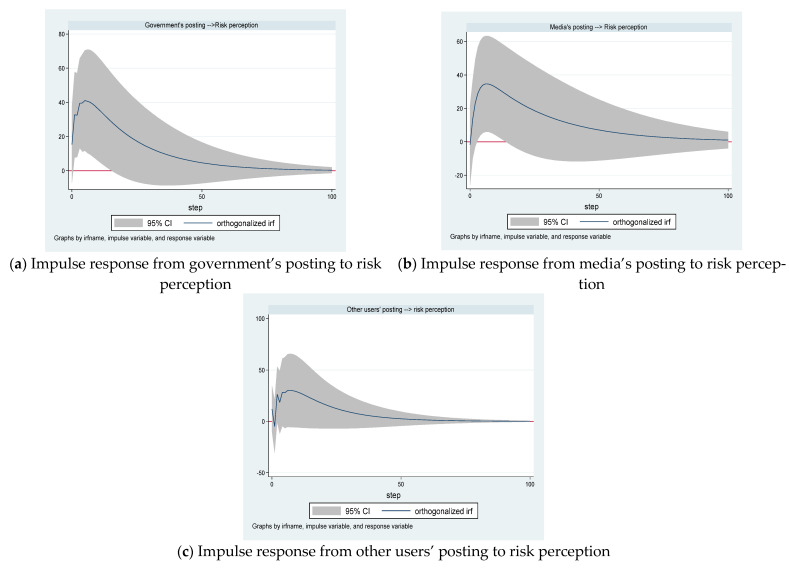
The dynamic effects of the postings on risk perception.

Figure 5a–c illustrates the reaction of risk perception to one standard deviation shock in postings from the government, the media, the public, and other users, respectively. The horizontal axis represents the lag period (unit: days), and the vertical axis represents the degree of change in risk perception. The solid line represents the impulse response function, which represents the response of risk perception to the impact of each corresponding variable.

Figure 5a shows that the effect of one standard deviation shock in the growth of the government’s posting on risk perception growth is positive. The maximum positive impact occurs on the fifth day with a value equal to 41.0999%. As the forecast period increases, the volatility of risk perception becomes smaller and smaller. From the sixteenth day onwards, the impact of changes in the amount of the government’s posting on risk perception growth is not significant.

The effect of one standard deviation shock in the growth of the media’s posting on risk perception growth is instantaneously negative but then positive from the first year (Figure 5b). The results show also that the maximum positive impact occurs on the sixth day. From the third day to the thirteenth day, the impact of changes in the volume of media’s posts on risk perception growth is significant, and it is not significant at other times.

The effect of one standard deviation shock in the growth of other users’ posting on risk perception growth is not significant (Figure 5c).

Hence, we can derive that the effect of one standard deviation shock in the growth of the government’s posting and the media’s posting on risk perception growth is significant and positive, but the effect of one standard deviation shock in the growth of other users’ posting on risk perception growth is not.

#### 4.2.2. Risk Perception Affecting Social Media Users’ Posting

**Figure 6:**
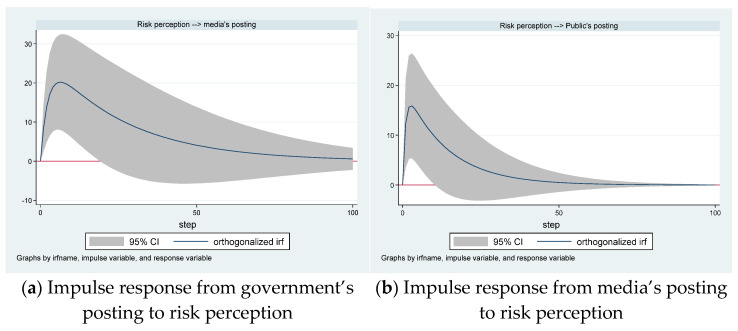
The dynamic effects of risk perception on the postings.

Figure 6a,b shows the reaction of media’s posting and public’s posting growth to one standard deviation shock in risk perception.

The effect of one standard deviation shock in the growth of risk perception on the growth of the media’s posting is positive (Figure 6a). The maximum positive impact also occurs on the sixth day. From the twentieth day, the impact of changes in risk perception on the growth of the media’s posting is not significant.

Figure 6b shows that the effect of one standard deviation shock in the growth of risk perception on public’s posting growth is positive. The maximum positive impact occurs on the third day. From the eleventh day onwards, the impact of changes in risk perception on the growth of the public’s posting is not significant.

The effects of one standard deviation shock in the growth of risk perception on the public’s posting growth and the media’s posting growth are significant and positive. Effective risk communication can motivate the public to carry out recommended actions.

## 5. Discussion

This research empirically shows the interplay between risk perception and social media posting in the Wuhan COVID-19 case.

### 5.1. Social Media Users’ Posting Affecting Risk Perception

Using Granger causality analysis, we found a unidirectional causality running from the government’s posting to risk perception, a bidirectional causality between the media’s posting and risk perception, and a unidirectional causality running from other users’ posting to risk perception. The results of impulse response functions indicate the effect of one standard deviation shock in the growth of the government’s posting and the media’s posting on risk perception growth is significant and positive, but the effect of one standard deviation shock in the growth of other users’ posting on risk perception growth is not. This means that the government’s posting and the media’s posting may affect the public’s risk perception, but other users’ posting may not affect the public’s risk perception.

The above conclusion is consistent with the proposal of Chan et al. [3] that the changes in the volume of information in social media are followed by different changes in risk perception. However, this result is not consistent with the conclusion of Xu, Qiu, Gu, and Ge’s [23] proposal that changes in risk perceptions are followed by changes in social media posting behavior in emerging infectious disease events. This means that in the Wuhan COVID-19 case, the role of posting by different users is diverse. In this case, it cannot be simply said that changes in risk perceptions are followed by changes in social media posting behavior or changes in the volume of posts in social media are followed by changes in risk perception.

This result may due to the reason that when new information appears due to the emerging of infectious disease threats, individuals must update their assessment of the risk [46]. Trust in the government and trust in media are significant factors in shaping the public’s risk perceptions of the virus, which in turn influence their intentions to engage in activities [47]. Such an understanding can enhance risk communication by making it more effective in giving people appropriate risk perceptions and motivating them to carry out recommended actions [4]. Therefore, it is very important for the government and the media to adopt appropriate communication strategies, use appropriate communication statements, and deliver appropriate risk information to the public for the control of the epidemic.

The quality of posts on social media is uneven. Early in the start of the pandemic, global leaders called upon the public to reject infodemics and access official sources [48]. Social media, especially the government and the media on social media, can be a tool for controlling rumors, providing regular updates, and fostering community cohesion in public health crises. These measures may affect the public’s risk perception and public protection behavior and then affect the progress of the pandemic.

### 5.2. Risk Perception Affecting Social Media Users’ Posting

We found a bidirectional causality between the media’s posting and risk perception and a unidirectional causality running from risk perception to the public’s posting. The effects of one standard deviation shock in the growth of risk perception on media’s posting growth and public’s posting growth are positive and significant. This means that risk perception may affect the public’s posting and the media’s posting.

The existence of a causality from risk perception to the public’s posting and the media’s posting is consistent with the conclusion of Xu et al.’s [23] proposal that risk perceptions have a dominant and immediate impact on SNS sharing behavior in emerging infectious disease events. However, it is not consistent with the proposal of Chan et al. [3]. This also shows the diverse relationship between different users’ posting and risk perception.

It is widely accepted that high risk perception will lead to personal preparedness and risk mitigation behavior. However, the relationship between risk perception and preparedness for actions is complex. The risk perception paradox exists in natural disasters; i.e., individuals with high risk perception still choose not to personally prepare themselves in the face of a natural hazard [22]. Is there a risk perception paradox in infectious disease events? The public has generally shown psychological reactions such as anxiety, depression, and panic in the COVID-19 pandemic. Perceiving the severity of the epidemic will increase the possibility of anxiety and fear. The study of Janis and Feshbach [49] showed that too high a level of anxiety in health messages is ineffective. On the other hand, there are many studies that confirm that behavior is more likely to change when messages have aroused strong anxiety [50]. Nevertheless, Leventhal, Zimmerman, and Gutmann [51] have shown that information about health risks alone does not change behavior, even if the information is received and understood. Therefore, can we assume that there is also a paradox of risk perception in pandemic events? Can this paradox of risk perception be explained by anxiety or fear? We continue the discussion in Section 5.3.

### 5.3. Limitation and Future Work

The method proposed in the paper can easily be used in other disaster events, but the results obtained may not be universal. In fact, the public’s risk perception and behavior are affected by the type of disaster, which may cause the relationship between risk perception and social media posting behavior in other disaster events to be inconsistent with the conclusions of this paper.

Social media communication is bidirectional (i.e., posting and reception). It is important to consider the bidirectional nature of social media communication in assessing the impact on risk perceptions [43]. Studies have shown that reception on social media is positively related to forming risk perceptions [17,26,27]. This paper only focuses on analyzing the relationships between the posting of different users and risk perception from the perspective of posting.

Our analysis is based on social network data in Wuhan from the end of 2019 to 26 April 2020. After that, a second or even third wave of epidemics occurred in some countries in the world. An obvious feeling is that in the subsequent epidemics, the public will pay relatively little attention to COVID-19. This may be because the public has adapted to the epidemic and is tired of related risk information. So, in the second or third wave of the epidemic, will the posting of the government and the media still affect the public’s risk perception? Is there any difference in the degree of influence compared with the first wave? These are the research questions we need to consider in the next step.

However, previous studies have shown that there are scenarios where the public ignores the epidemic information. According to Davison’s third-person hypothesis [52], people are optimistically biased and would perceive others as being more vulnerable than themselves [53]. Increased psychological distance may lead individuals to view the risk more abstractly and discount it more heavily than those who perceive the risk as psychologically proximal and concrete [54]. So, will the difference in actual distance or psychological distance cause the changes in these relationships discussed in this article? This is also a research question worth discussing.

As mentioned earlier, there may be a risk perception paradox. Can we analyze users’ emotions, especially anxiety and fear, by analyzing the content of users’ posts on social media so as to further discuss the direction of the relationships of risk perception and the public’s behavior (i.e., positive or negative influence, or inconsequential)? This is another research question worth studying.

## 6. Conclusions

COVID-19 has been a huge challenge for all countries in the world. During a health emergency, receiving timely and accurate information enables individuals to take appropriate actions to protect themselves, shaping their risk perception [55]. Dividing users on Sina Weibo into the government, the media, the public, and other users, we explored the interplay between risk perception and social media posting of different users through a case study of COVID-19 in Wuhan.

From one perspective, the government and the media on Sina Weibo play critical roles in forming and affecting risk perceptions during COVID-19 in Wuhan. The increase of the government’s posting and the media’s posting can significantly enhance the public’s perceptions of risk issues. Thus, the government and the media must remain vigilant to provide credible risk-related information. From another perspective, risk perception promotes the posting of the media and the public on Sina Weibo. Effective risk communication can motivate the public to carry out recommended actions.

## Figures and Tables

**Table 1:** The relationship between social media posting and risk perception.

No.	Conclusions and Reference	Data Sources
1	Social media posting correlated with the level of risk perception Changes in the volume of information in social media are followed by changes in risk perception [3].	Twitter and Questionnaire
2	Changes in risk perceptions are followed by changes in social media posting and reposting behavior [23].	Sina Weibo
3	Posting information on social media is positively associated with risk perceptions [43].	Questionnaire
4	Posting and receiving risk information not only affected risk perceptions but also directly or indirectly influenced preventive behavioral intentions [35].	Questionnaire

**Table 2:** Descriptive statistics of social media data.

Type of Users	Number of Users	Number of Microblogs	Average Number of Microblogs Per User	Minimum Number of Microblogs Per User	Maximum Number of Microblogs Per User
Government	292	4900	16.78	1	840
Media	237	28,985	122.30	1	8208
Public	14,017	31,552	2.25	1	589
Others	661	6971	10.55	1	1731

**Table 3:** Definition of variables.

Variable Type	Variable	Definition
Endogenous variables	$Perception_{t}$	Risk perception, expressed by Baidu Search Index within time window t
	$Posting^{0}$	The volume of government’s posting within time window t
	$Posting^{1}$	The volume of media’s posting within time window t
	$Posting^{2}$	The volume of public’s posting within time window t
	$Posting^{3}$	The volume of other users’ posting within time window t
Exogenous variables	NewCases	The number of new cases within the time window t

**Table 4:** Results of Granger causality tests between government, media, public, and other users’ posting and risk perceptions.

Null Hypothesis	Lag Length	F-Value, *p*-Value	Results
Risk perception is not the Granger reason for government’s posting.	Two days	3.523, 0.172	Accept
Government’s posting is not the Granger reason for risk perception.	Two days	6.5978, 0.037	Reject
Risk perception is not the Granger reason for media’s posting.	One day	8.2307, 0.004	Reject
Media’s posting is not the Granger reason for risk perception.	One day	5.0754, 0.024	Reject
Risk perception is not the Granger reason for public’s posting.	One day	7.8697, 0.005	Reject
Public’s posting is not the Granger reason for risk perception.	One day	0.05545, 0.814	Accept
Risk perception is not the Granger reason for other users’ posting.	Two days	4.1178, 0.128	Accept
Other users’ posting is not the Granger reason for risk perception.	Two days	7.7616, 0.021	Reject

## Data Availability

The data presented in this study are available on request from the corresponding author. The data are not publicly available due to the restrictions of the social media platform.
